# Sensorineural Hearing Loss After Transvenous Embolization of a Transverse-Sigmoid Sinus Dural Arteriovenous Fistula

**DOI:** 10.7759/cureus.107255

**Published:** 2026-04-17

**Authors:** Kensaku Yoshida

**Affiliations:** 1 Department of Neurosurgery, Tokyo Metropolitan Hiroo Hospital, Tokyo, JPN

**Keywords:** cerebral venous congestion, dural arteriovenous fistula, sensorineural hearing loss, transvenous embolization, transverse sigmoid sinus

## Abstract

We report a case of sensorineural hearing loss following transvenous embolization (TVE) of a transverse-sigmoid sinus dural arteriovenous fistula (TS-DAVF) with cortical venous reflux from an isolated sinus. A 64-year-old woman was transferred to our hospital on an emergency basis due to slurred speech and suspected stroke. Magnetic resonance imaging (MRI) revealed TS-DAVF with cortical venous reflux on the left side. We diagnosed her with TS-DAVF and Cognard type IIa+b, and performed TVE via an occluded sigmoid sinus. Posttreatment angiography confirmed the absence of an arteriovenous shunt.

On the day after treatment, she experienced dizziness and nausea upon movement, and was diagnosed with inner ear vertigo, for which medical treatment was initiated. Postoperative MRI showed no ischemic lesions on diffusion-weighted imaging and revealed shrinkage of dilated cortical veins on T2-weighted imaging. However, on the seventh postoperative day, she noticed hearing loss on the left side. An otolaryngological examination confirmed sensorineural hearing loss, and she was subsequently treated with steroids. Her hearing impairment gradually improved, and as the hearing loss did not interfere with her daily life, she was discharged on the 12th postoperative day.

There are several case reports of sensorineural hearing loss following TVE for TS-DAVF, indicating this is a potential complication that should be considered, especially when there is extensive occlusion of an isolated sinus.

## Introduction

Dural arteriovenous fistulas (DAVFs) are abnormal shunts between dural arteries and venous sinuses or cortical veins, with clinical presentations ranging from asymptomatic to aggressive neurological deficits, including hemorrhage or venous infarction. The transverse-sigmoid sinus is the most common location of DAVFs, and endovascular treatment, including transvenous embolization (TVE) and transarterial embolization, is widely used as the first-line therapeutic approach. TVE is a widely recognized and effective treatment for transverse-sigmoid dural arteriovenous fistula (TS-DAVF), a rare and potentially dangerous vascular condition. While TVE is generally effective, it carries risks; common complications include venous infarction due to venous obstruction and bleeding. The transient complication rate for TVE has been reported as 10%-42%, with permanent morbidity rates of 4%-5% [1-3]. Hearing loss, though infrequently reported, has emerged as a rare but noteworthy complication of TVE for TS-DAVF.

Here, we present a case of sensorineural hearing loss following TVE in a patient with TS-DAVF and cortical venous reflux from an isolated sinus, highlighting a potential complication that may require further attention in clinical practice.

## Case presentation

A 64-year-old woman experienced a transient episode of impaired tongue movement and slurred speech lasting approximately five minutes. Although her symptoms resolved spontaneously, she was admitted to our hospital with a suspected stroke. Her medical history included lacunar infarction and paroxysmal atrial fibrillation, with no known allergies. Upon arrival, her neurological examination was unremarkable, as her dysarthria had resolved. She displayed no hearing impairment, facial nerve palsy, or subjective vascular murmur.

Imaging findings

**Figure 1:**
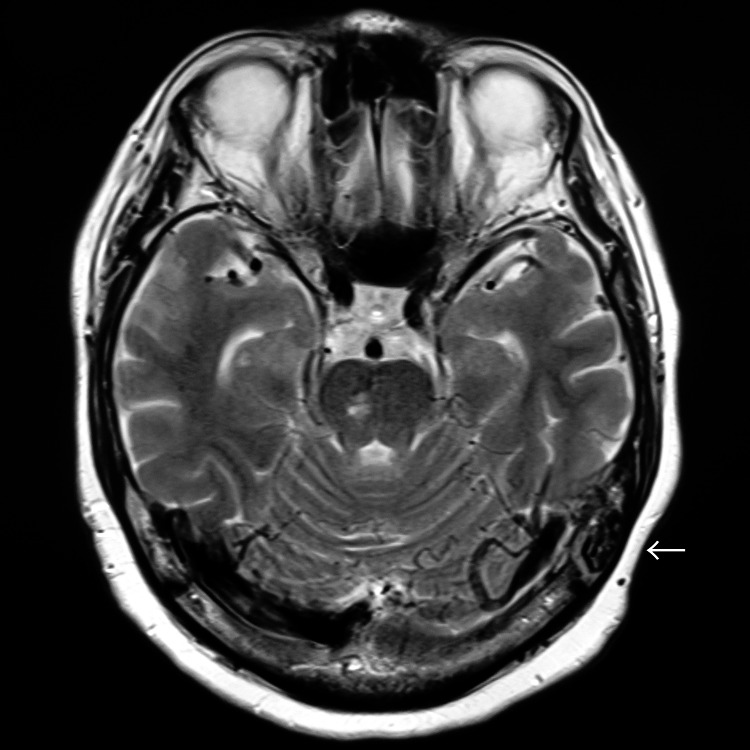
MRI T2-weighted image showing multiple dilated veins gathered around the left transverse sinus (white arrow) MRI: magnetic resonance imaging

**Figure 2:**
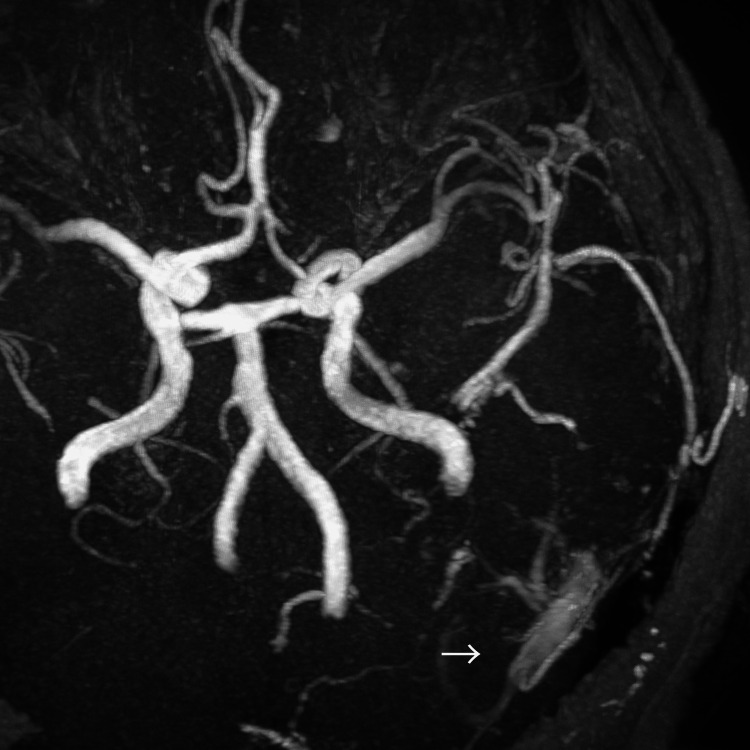
MRA showing dilated vessels at the left transverse sigmoid junction (white arrow) MRA: magnetic resonance angiography

**Figure 3:**
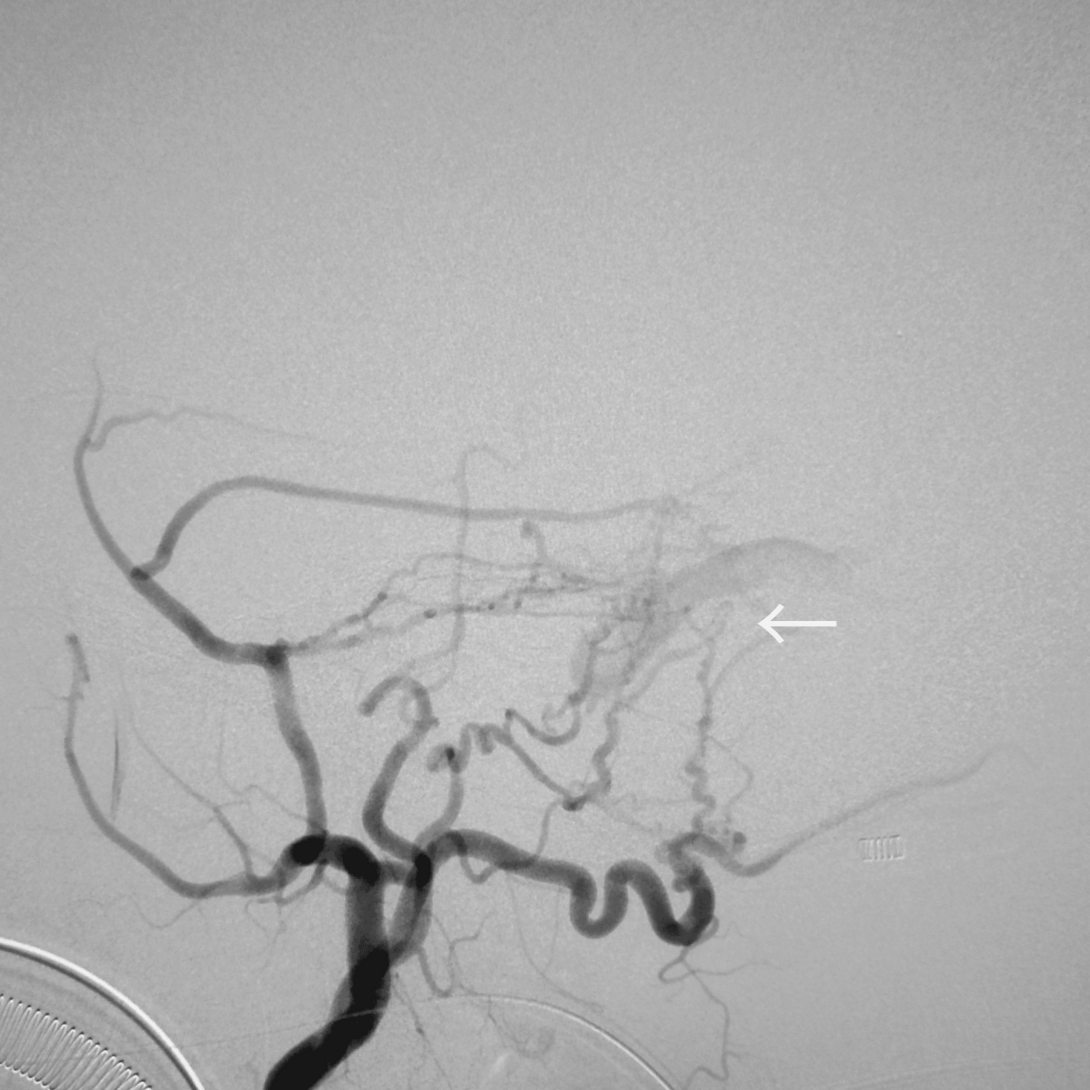
Lateral view of the left external carotid artery angiogram showing the left TS-DAVF in the arterial phase (white arrow) TS-DAVF: transverse-sigmoid sinus dural arteriovenous fistula

**Figure 4:**
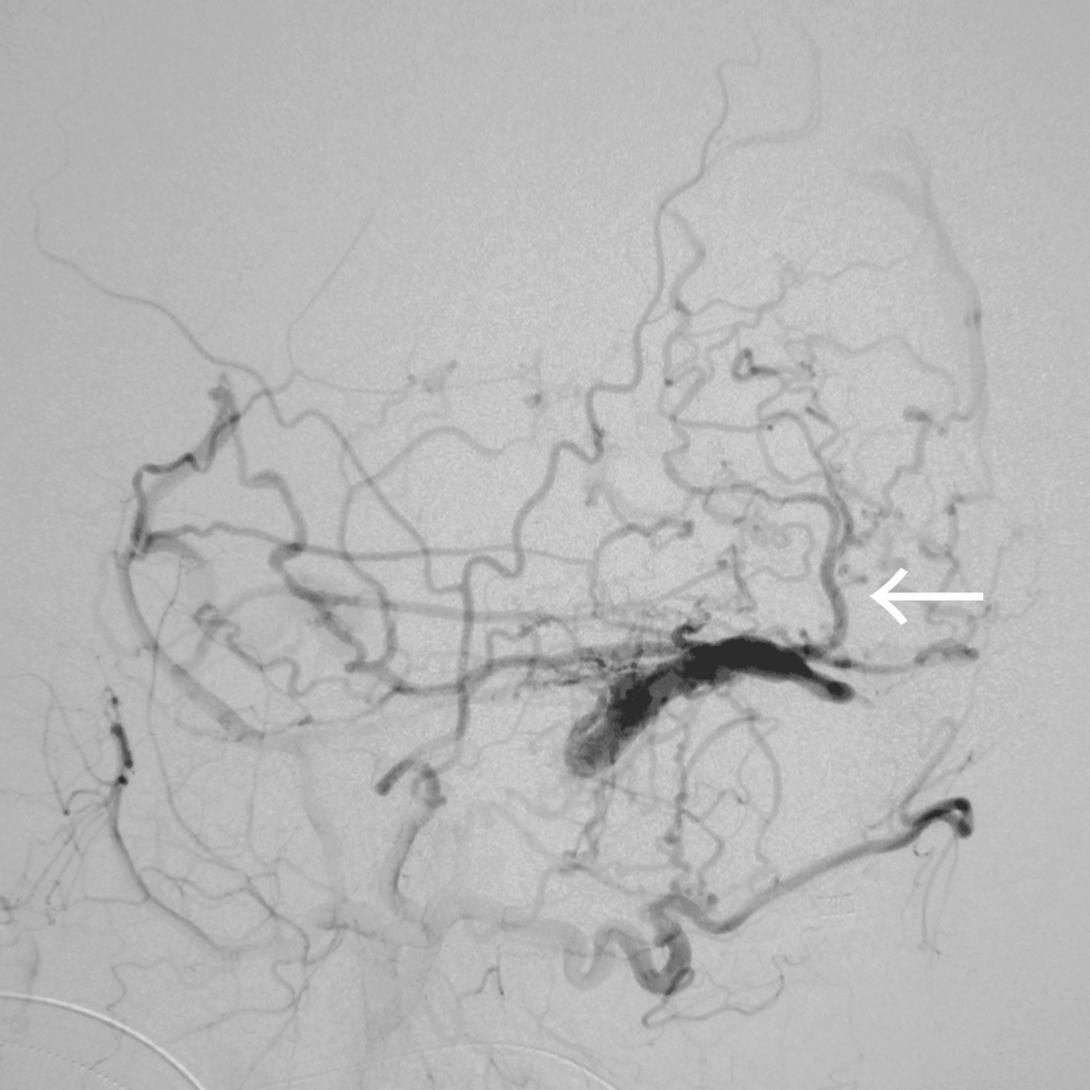
Lateral view of the left ECA angiogram showing an obvious retrograde cortical venous reflux in the late arterial phase (white arrow). No prominent left-sided vein of Labbe was identified on angiography ECA: external carotid artery

Head magnetic resonance imaging (MRI) revealed ischemic changes in the cerebral white matter and basal ganglia, multiple microbleeds, and dilated cerebral veins in the left occipital lobe and left cerebellum (Figure 1). MRA showed dilated vessels at the left transverse-sigmoid junction (Figure 2). A left external carotid artery angiogram demonstrated a left TS-DAVF (Figures 3, 4). Feeding arteries included the left middle meningeal artery, occipital artery, and superficial temporal artery, with drainage into an isolated sinus and retrograde reflux into the left occipital cortical veins and superior petrosal sinus (SPS). Drainage in the right occipital and parietal lobes was observed via the superior sagittal sinus and internal occipital vein.

Based on these findings, we diagnosed TS-DAVF (Cognard type IIa+b, Borden type II) and decided to perform TVE. TVE was selected because the isolated sinus could be accessed from the affected side, allowing occlusion of the dangerous drainage pathway.

Procedure

**Figure 5:**
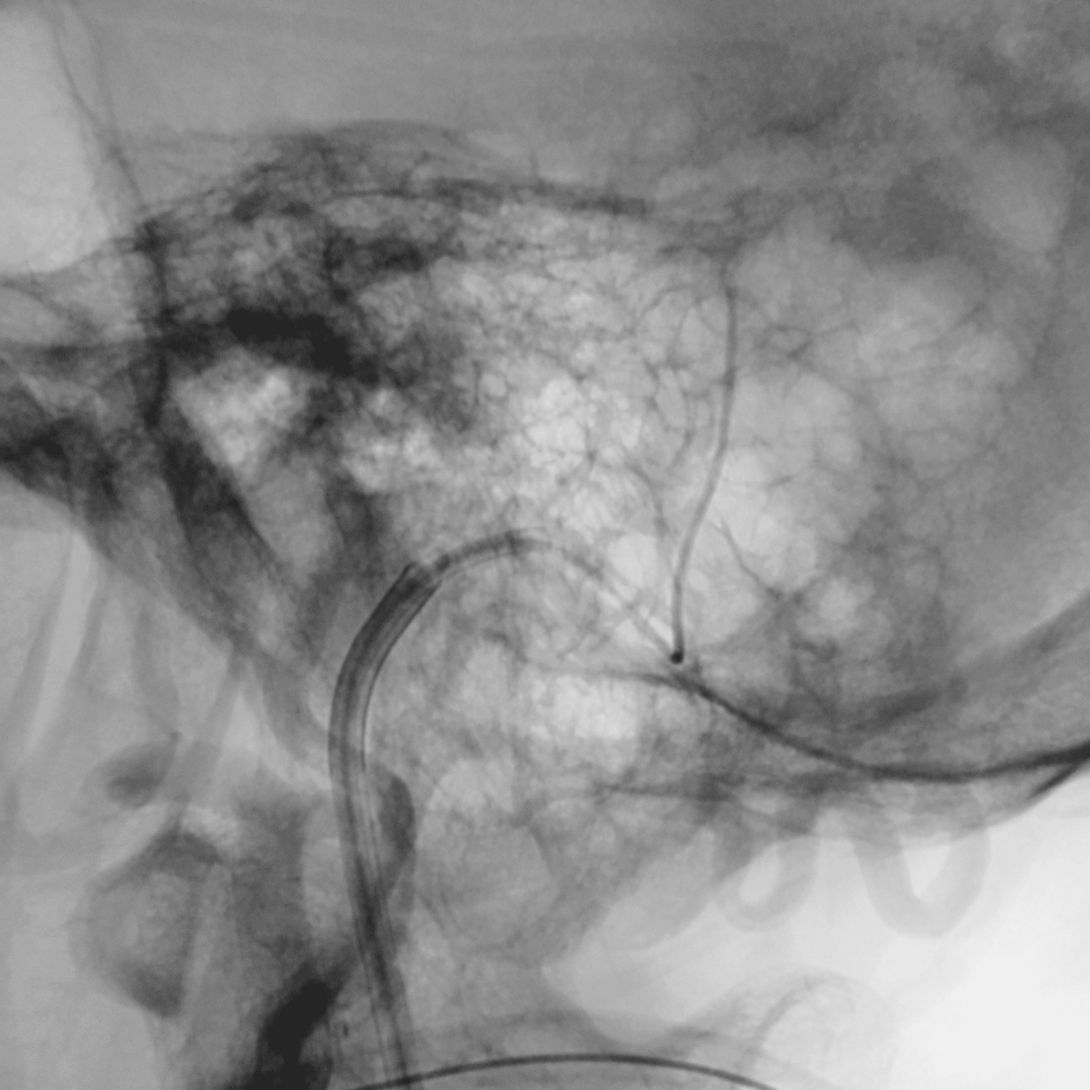
Approach route showing the catheter navigating into the isolated sinus

**Figure 6:**
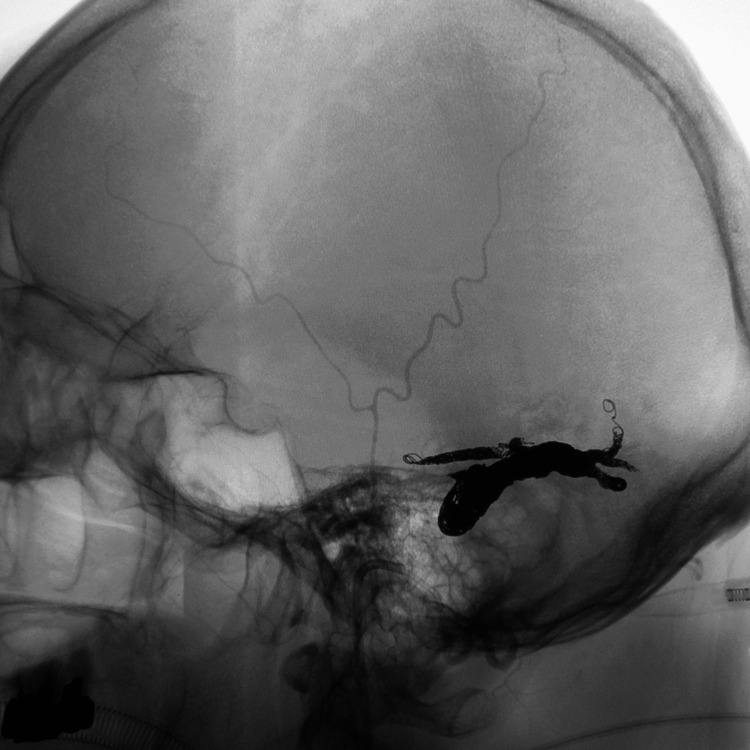
Lateral view of craniogram after embolization, recognizing inserted coils in the draining veins and sinus

**Figure 7:**
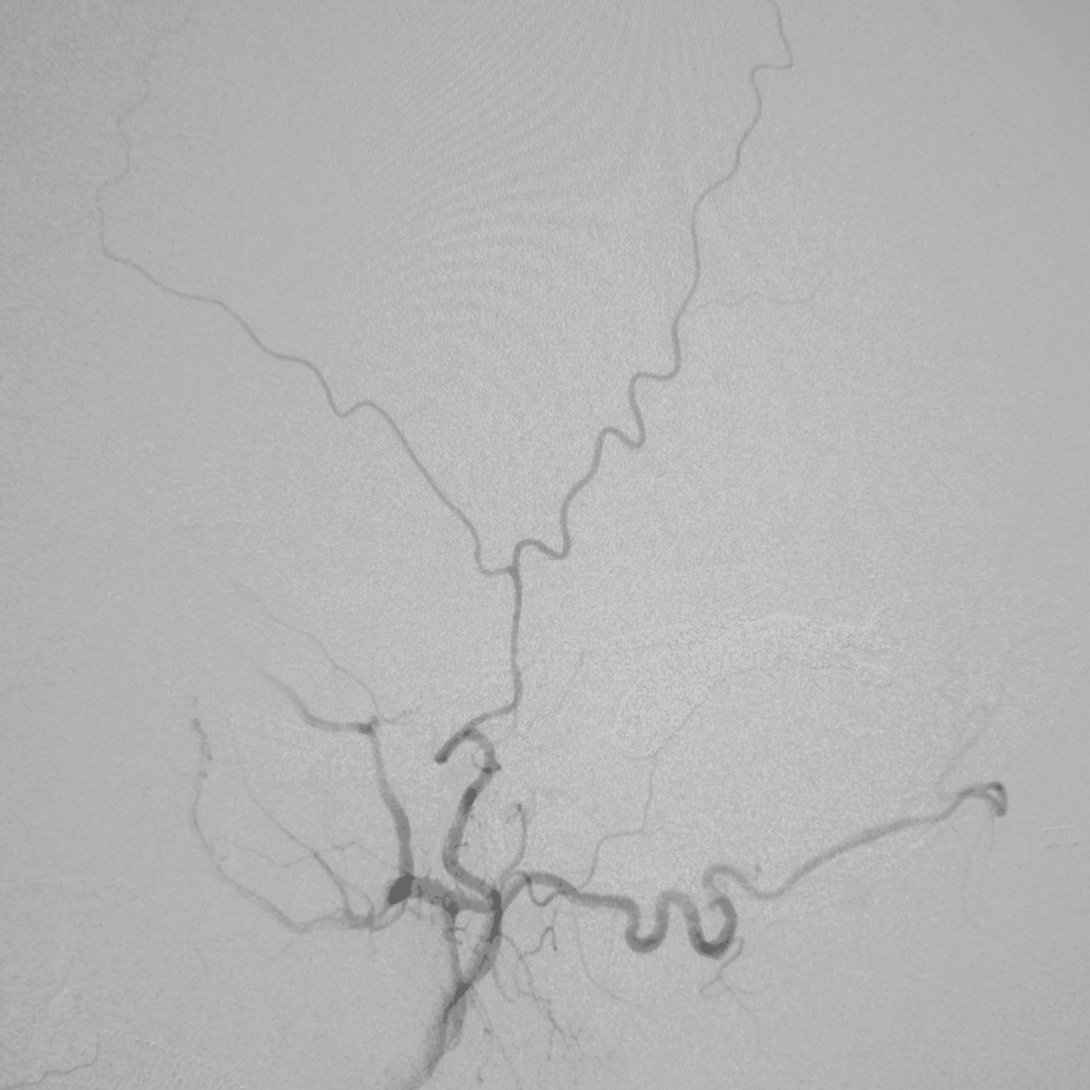
Lateral view of the left ECA angiogram after TVE showing no arteriovenous shunt after embolization ECA: external carotid artery; TVE: transvenous embolization

Under general anesthesia, a 6-Fr long sheath was inserted into the right femoral vein. An Envoy 6 Fr MPC 90 cm (Johnson & Johnson, New Brunswick, NJ) and a Medikit 4 Fr 106 cm (Medikit, Tokyo, Japan) catheter were advanced from the left internal jugular vein to the isolated sinus (Figure 5). Since the contralateral venous sinus was patent, its structure was used as a reference, and the occluded vein was carefully perforated with a guidewire while observing the skull base structure on X-ray. A microcatheter was navigated to the isolated sinus, where the left SPS, cortical veins, and isolated sinus were occluded using 51 coils (Figure 6). The operation involved 160 mL of contrast medium, with a duration of five hours and 46 minutes, a fluoroscopy time of 169 minutes, and a radiation dose of 3,243 mGy. Posttreatment angiography confirmed no residual arteriovenous shunt (Figure 7). During the procedure, activated clotting time was maintained at 2-2.5 times the baseline level, and systemic heparinization continued for two days postoperatively.

Postoperative course

Postoperatively, the patient reported discomfort in the left occipital region and experienced dizziness and nausea upon movement. She was diagnosed with left-sided inner ear vertigo and began medical treatment. Anticoagulation therapy was maintained to prevent thrombus formation due to delayed cortical vein perfusion, and she was switched to direct oral anticoagulants for her atrial fibrillation. Follow-up MRI showed no ischemic lesions on diffusion-weighted imaging, and T2-weighted imaging demonstrated a reduction in the dilated cortical veins, including the absence of brainstem or internal auditory canal abnormal lesions.

While her vertigo symptoms gradually improved, she developed left-sided hearing loss on the seventh postoperative day. An otolaryngological evaluation diagnosed sensorineural hearing loss, and she was started on steroid therapy. Her audiogram indicated postoperative hearing thresholds of 11.3 dB on the right and 37.5 dB on the left, despite no preoperative hearing complaints.

Her hearing gradually improved with steroid treatment. Given the mild degree of hearing loss, which did not interfere with daily activities, she was discharged on the 12th postoperative day.

## Discussion

This case describes a patient who developed ipsilateral sensorineural hearing loss following TVE for a Borden type 2 TS-DAVF with retrograde cortical venous drainage. TVE for TS-DAVF involves occlusion of affected veins and sinuses to eliminate multiple shunts, and hearing loss or vestibular symptoms have been reported following this procedure, primarily in female patients who underwent sinus packing (Table 1).

**Table 1:** Patients characteristics with dizziness after TVE for TS-DAVFs SS: sigmoid sinus; TVE: transvenous embolization; DC: detachable coils; NA: not available; POD: postoperative day; GOS: Glasgow Outcome Scale; GR: good recovery; TS-DAVF: transverse-sigmoid sinus dural arteriovenous fistula

Case no.	Age	Sex	Location	Borden	Cognard	Clinical presentation	Procedure	Material	POD	Treatment	Duration	GOS	Reference
1	59	F	Perpendicular of SS	2	Ⅱa+b	Hearing disturbance	TVE	DC	3	NA	NA	NA	[1]
2	65	F	Transsigmoid junction	1	Ⅰ	Hearing disturbance	TVE	DC	3	Steroid	6 months	GR	[1]
3	72	F	Perpendicular of SS	2	Ⅱa+b	Hearing disturbance	TVE	DC	10	NA	2 weeks	GR	[4]
4	64	F	Transsigmoid junction	2	Ⅱa+b	Hearing disturbance	TVE	DC	7	Steroid	12 days	GR	Present case

Identifying contributing factors to post-TVE sensorineural hearing loss is challenging, but previous case reports provide some insights [5-7]. In this case, vestibular symptoms appeared immediately posttreatment and progressively transitioned to hearing loss. Potential causes of sensorineural hearing loss include endolymphatic edema from venous thrombosis, radiation effects, contrast media, and anesthesia.

Several authors have discussed similar cases. Kuwayama et al. observed endolymphatic edema, suspecting venous occlusion affecting the inner ear due to sinus packing [8]. Yamauchi et al. highlighted the possibility of inner-ear venous thrombosis and paradoxical worsening after TVE for cavernous sinus DAVF [4]. Roy and Raymond hypothesized a link between postoperative hearing loss and endolymphatic edema, although a direct causal relationship remains unproven [9].

Anatomical factors could also contribute. The vein of the vestibular aqueduct drains into the sigmoid sinus, while the inferior cochlear vein drains into the inferior petrosal sinus (IPS). In this patient, IPS occlusion may have impaired venous drainage, potentially affecting the inner ear and resulting in dizziness and hearing loss due to thrombosis of the vestibular aqueduct.

Alternative approaches, such as TVE without sinus packing, could mitigate risks to the inner ear. Terada et al. described the use of balloon protection of the sinuses during transarterial embolization for Borden types 1 and 2 DAVFs to improve venous drainage [10]. The use of liquid embolic agents could have reduced procedure complexity, coil number, and radiation exposure.

Other factors that might have contributed to hearing loss include radiation exposure, contrast media, and general anesthesia. Bhandare et al. reported that radiation doses exceeding 60.5 Gy significantly increased the risk of sensorineural hearing loss [5]. Contrast-induced chemotoxicity or delayed allergic reactions have been implicated in hearing loss after angiographic procedures [6]. Additionally, microemboli from general anesthesia, particularly during surgeries such as cardiopulmonary bypass, as well as increased middle ear pressure from nitrous oxide or the Valsalva maneuver, are known to cause cochlear damage [7].

In cases of sudden hearing loss, steroid and low-molecular-weight dextrin therapy can promote recovery. In this case, steroid administration improved both vestibular and cochlear symptoms. Prompt recognition and intervention are essential, as mild sensorineural hearing loss often resolves or improves with early treatment.

## Conclusions

Early detection and treatment are important in cases of the postoperative complication of sensorineural hearing loss after TVE of a TS-DAVF. In this case, isolated sinus occlusion with TVE was thought to have caused endolymphatic edema, resulting in inner ear circulatory disturbance.
